# Emotional cue validity effects: The role of neurocognitive responses to emotion

**DOI:** 10.1371/journal.pone.0179714

**Published:** 2017-07-06

**Authors:** Samantha Denefrio, Akeesha Simmons, Amishi Jha, Tracy A. Dennis-Tiwary

**Affiliations:** 1 The Graduate Center, The City University of New York, New York, New York, United States of America; 2 Hunter College, The City University of New York, New York, New York, United States of America; 3 University of Miami, Miama, Florida, United States of America; Vanderbilt University, UNITED STATES

## Abstract

The beneficial effect of valid compared to invalid cues on attention performance is a basic attentional mechanism, but the impact of emotional content on cue validity is poorly understood. We tested whether the effect of cue validity on attention performance differed when cues were angry, happy, or neutral faces. Moreover, we used scalp-recorded event-related potentials (ERPs) reflecting the capture of early visual attention (P1, N170) to test whether effects were strengthened when neurocognitive responses to angry or happy cues were enhanced (larger P1 and N170 amplitudes). Twenty-five participants completed a modified flanker task using emotional face cues to measure the effects of emotion on conflict interference. Attention performance was enhanced following valid versus invalid cues, but effects did not differ by emotion cue type. However, for participants showing relatively larger N170 amplitudes to angry face cues, attention performance was specifically disrupted on those trials. Conversely, participants with relatively larger N170 amplitudes to happy face cues showed facilitated performance across all valid trials. These findings suggest that individual neurocognitive sensitivities to emotion predict the impact of emotional content on the basic attentional phenomenon of cue validity.

## Introduction

The effect of cue validity on attention performance is a well-studied phenomenon within the literature on spatial attention. Valid cues are those that give accurate information on the location of a target, whereas invalid cues provide misleading information and detract from the ability to predict the location of a target [1]. Compared to invalid cues, valid cues confer a performance advantage, including more efficient attention performance [2,3] and shorter reaction times [4] particularly when participants willfully attend to a valid cue location [5].

While the influence of cue validity is widely accepted, relatively little is known about contextual factors that may influence cue validity effects. One such context is emotion, but previous research provides conflicting evidence concerning whether the valence of emotional stimuli—positive or negative—facilitates or disrupts cue validity effects [6, 7, 8]. Moreover, it is unclear whether individual differences in the degree to which emotional stimuli capture attention influences the effects of emotion on cue validity. The goals of the current study were to (a) examine the impact of emotion on cue validity effects during a conflict interference task; and (b) use a neurocognitive measure of early visual processing—scalp-recorded event-related potentials—to examine whether emotion by cue validity effects are heightened when individuals show greater neurocognitive responses to emotional cues.

Theories of the impact of emotion on attention are highly relevant to the question of whether and how emotion influences cue validity effects. These theories have traditionally focused on the differential impact of positive versus negative emotions. According to Easterbrook [9], negative emotions not only recruit attention but also aid the processing of relevant cues by constraining perception [10]. A number of studies support the premise that negative emotions initially narrow the scope of visual attention by effectively engaging the participant in goal-directed behavior [11, 12]. Yet, other studies have documented a disruption in performance on tasks that require narrowed attention such as when participants are asked to count facial features of negative face stimuli [13] or to determine if two presented stimuli are the same or different when negative faces are simultaneously presented in unattended locations [14].

The broaden-and-build theory proposes that negative and positive emotions have divergent cognitive and attentional correlates [15, 16]. Positive emotions have the ability to expand attentional focus resulting in a more global attention bias and an increased awareness of distal information on global-local visual processing tasks [17, 18, 19] broadened thinking, creativity [20, 21, 22] and greater distractibility [23]. Yet, counter to this formulation, positive mood inductions have also been shown to effectively focus attention towards a target and away from peripheral distractors [24].

Furthermore, it has been suggested that positive emotions may narrow attentional focus if the individual’s approach motivation is high [25, 26, 27, 28]. For example, opposing affective states such as anger or enthusiasm that are similarly high in intensity are motivating and should direct attention towards a goal. In two studies, induction of a narrowed scope of attention enhanced sensitivity to both appetitive and aversive stimuli but not to neutral pictures as measured by the N1 event-related potential [29]. Together, these findings suggest that both positive and negative emotions can lead to similar cognitive performance (i.e., narrowed attention).

In an attempt to account for these findings that diverge from traditional models of emotion and attention, Clore & Huntsinger [30, 31] proposed the affect-as-information model in which the influence of emotion on attention is flexible rather than fixed, with positive emotion acting like a “go signal” for whatever attentional focus is currently dominant, and negative emotion acting like a “stop signal.” Therefore, if completing a task that activates the narrowing of attention such as a flanker task, then positive emotion should facilitate this narrowing of attention to promote task performance. In contrast to theories arguing that negative emotion serves to narrow attention, in the context of a task in which narrowed attention is dominant, the presence of negative emotion will inhibit narrowing and instead serve to broaden attention.

A range of studies is consistent with this recent formulation [32]. For example, Fenske & Eastwood [33] used a modified flanker task with emotionally-salient faces in place of the traditional directional arrows. A face expressing either positive or negative emotion was flanked by faces of either the same “compatible” or opposite/neutral “incompatible” emotion. The task required participants to identify the emotion expressed by the face located in the center. Overall, reaction times were fastest on compatible trials, specifically when the target emotion was positive, suggesting that positive affect facilitated the dominant mode of attention (narrowed) and enhanced performance. Further supporting their model, Huntsinger [34] found that when individuals were primed with either a local or global focus of attention and completed mood induction tasks, the positive versus negative mood induction facilitated the primed focus. Specifically, when a narrowed focus was primed, the positive mood induction improved flanker performance, consistent with the notion that facilitation of a narrowed focus of attention reduces incompatible flanker interference. Conversely, when a global focus was primed, this effect was reversed such that the positive mood induction reduced flanker performance.

Research combining emotional content and cue validity can further help to reveal how emotion affects predicted attention performance. In a series of experiments, Fox et al. [35] found that when high state-anxious participants were presented with angry faces as valid and invalid cues, as compared to happy and neutral faces, reaction times to identify the spatial location of target stimulus (white circle) were significantly longer than in the low anxious group. This suggests a specific disruption in a task requiring narrowed attention by negative emotional cues for the high anxious group only. Emotional cue type also influenced validity effects in the high state-anxious group: the largest differences in reaction time on valid compared to invalid cue trials followed angry cues. Taken together, these findings suggest that the role of emotion on attention performance may differ depending not only on task characteristics but on individual differences as well.

One important limitation to previous research on emotion and cue validity and on the impact of emotion on attention performance is that these studies have relied almost exclusively on behavioral metrics (i.e. reaction times) and priming conditions (i.e. mood induction) that lack the ability to track individual differences in responses to emotional stimuli beyond self-reported affective experiences. Instead, by examining more stable and enduring individual differences on the physiological level, it may be possible to glean insights into the degree to which emotion captures attentional resources to facilitate or inhibit subsequent task performance.

Scalp-recorded event-related potentials (ERPs), given their excellent temporal resolution and functional sensitivity to discrete cognitive processes, are particularly well-suited to measuring the time course of attention to emotion [36, 37, 38, 39, 40, 41, 42]. However, little is known about whether relatively early and automatic responses to emotional stimuli influence the impact of emotional content on attentional processes such as cue validity. Previous research has documented changes in a number of later-emerging ERPs, such as the P2, N2, P3, and ERN [43, 44, 45]. from attention assays like the flanker task. These studies show, for example, that degree of conflict interference during a cued flanker correlates with N2 amplitudes and trait anxiety [45]. In the current study, we examined ERPs that were both relatively early-emerging and sensitive to emotional faces—the P1 and N170, thus targeting finely-grained stages of early processing of and attention to visual emotional cues (angry and happy faces) and examining whether these rapidly-emerging neurocognitive responses predict cue validity effects.

The P1 is a positive-going ERP with a maximal peak occurring around 100 ms post stimulus over posterior regions of the scalp. The P1 reflects activity of the extrastriate area of the visual cortex [46]. That is, as a greater number of neurons are recruited, P1 amplitudes increase [47, 48, 49]. Larger P1 amplitudes have been associated with the rapid, global processing of low intensity stimuli [50] and with correctly directed spatial attention [51, 52]. The P1 is also enhanced to salient emotional faces [53, 54, 55] such as fearful compared to neutral faces [56, 57, 58]. Furthermore, validly cued targets increase Pl amplitudes reflecting attention-enhanced sensory processing [42]. Thus, the P1 is thought to be a relatively direct measure of early spatial attention and attention allocation.

A second early-occurring ERP, the N170, is a negative deflection occurring around 170 to 270 ms post stimulus. Larger N170 amplitudes occur in response to viewing faces versus objects [59] and when viewing face-specific parts such as a nose or eyes [60]. Thus, the N170 may reflect the degree of face-specific attention processing [61, 62]. While there is debate about the emotional sensitivity of the N170 [63, 64], a growing number of studies document that N170 amplitudes are larger to emotional versus neutral faces in adults [61, 65, 66, 54] and children [67] and that the emotional enhancement of the N170 predicts individual differences in emotional behavior [68].

The goal of the current study was two-fold. First, we examined the impact of emotional content on cue validity, which was measured behaviorally as the difference in reaction times during trials of a flanker task modified to include valid and invalid face cues. The flanker task was chosen because it requires a narrowing of attention (focused attention on the central arrow while ignoring incongruent flanking distracters). Consistent with Huntsinger [32], we predicted that the effects of cue validity would be enhanced when cues are emotionally positive (happy faces) because they facilitate (“go signal”) the dominant mode of narrowed attention resulting in enhanced performance, whereas, in contrast, effects of cue validity will be reduced when cues are emotionally negative (angry faces) because they will disrupt (“stop signal”) narrowed attention. Second, we examined face cue processing using ERPs. We predicted that these effects of emotion on cue validity will be heightened when individuals show greater neurocognitive responses (i.e., greater P1 and N170 amplitudes) to emotional cues. That is, individuals showing enhanced ERP responses to happy faces should be particularly sensitive to positive emotional cues and show significantly facilitated performance. Similarly, individuals showing enhanced processing of angry faces should evidence the greatest disruption in performance following angry cues.

## Method

### Participants

The Hunter College Internal Review Board (IRB) approved all stimuli, tasks, and procedures. Twenty-five adults (16 females) between the ages of 18 and 36 (*M* = 21.79, *SD* = 5.27) participated from an urban college in New York City. Of the 27 participants recruited for the study, two were unable to be included in analyses involving ERPs due to excessive movement artifacts during EEG recording. Additionally as a result of computer failure one participant did not have behavioral reaction time data. Therefore, the final sample for this study includes 24 participants with reaction time data and 25 participants with ERP data. In addition, demographic data was missing for one participant. Self-reported race/ethnicity for the 24 participants was as follows: 10 White/Caucasian, seven Asian, one Black/African American, two Native Hawaiian or Pacific Islander, one more than one race, and three “Other”.

### Stimuli and materials

The emotional face stimuli were taken from the NimStim database of the Research Network on Early Experience and Brain Development [69]. All photographs were approximately 177 x 228 pixels, and displayed in grayscale against a white background. The stimuli were equally divided between males and females. Ten actors were White/Caucasian and the remaining six were African American. Three photographs for each actor were used, portraying an angry, happy, and neutral expression for a total of 48 photographs of faces. (Actor Numbers used: Males—20, 21, 23, 30, 33, 38, 39, and 43; Females—01, 06, 07, 08, 10, 12, 13, 14).

The face-cued flanker task was presented on an IBM computer with a 17” monitor and was run using E-PRIME software (Psychological Software Tools, Pittsburgh, PA., version 1.1) Participants were seated approximately 65” away from the computer monitor during the task.

### Procedure

Following consent procedures, participants completed a series of questionnaires pertaining to demographic information. After questionnaires were finished, participants completed a modifed flanker task that uses emotional faces as valid/invalid cues, while EEG was continuously recorded. Each participant spent approximately two and a half hours in the laboratory to complete the study. The institution’s Internal Review Board (IRB) approved all stimuli, tasks, and procedures.

### Modified flanker task with valid and invalid face cues

This task was derived from previous studies using a modified face-cued flanker task [70, 6] called the Attention Network Test [71]. The flanker task that requires the subject to identify the direction (right or left) of the central target arrow that points toward either the left or right side of the screen. Participants are instructed to press a button to indicate the direction of the arrow (left or right). Additional arrows on both sides flank the target arrow. On some trials, the flanker arrows point in the same direction of the target; hence, these trials are referred to as “congruent” flankers. On other trials, the flankers point towards the opposite direction, and are thus referred to as “incongruent”. Cue validity effects were quantified behaviorally by subtracting responses to congruent flanker trials from responses to incongruent trials yields the conflict interference score. A higher score indicates greater cognitive conflict, or lower executive attention functioning.

For the cues, prior to each flanker, an emotional (angry, happy) or neutral face was presented for 100 ms. These face cues were presented in either a valid or invalid location relative to the subsequent flanker display. A valid cue appeared in the exact location where the flanker would appear (center, left, or right). In contrast, invalid cues appeared in one of the three possible locations where the flanker did not appear on a given trial. In addition, there were comparison trials with a cue in the center of the screen and no-cue trials.

**Fig 1:**
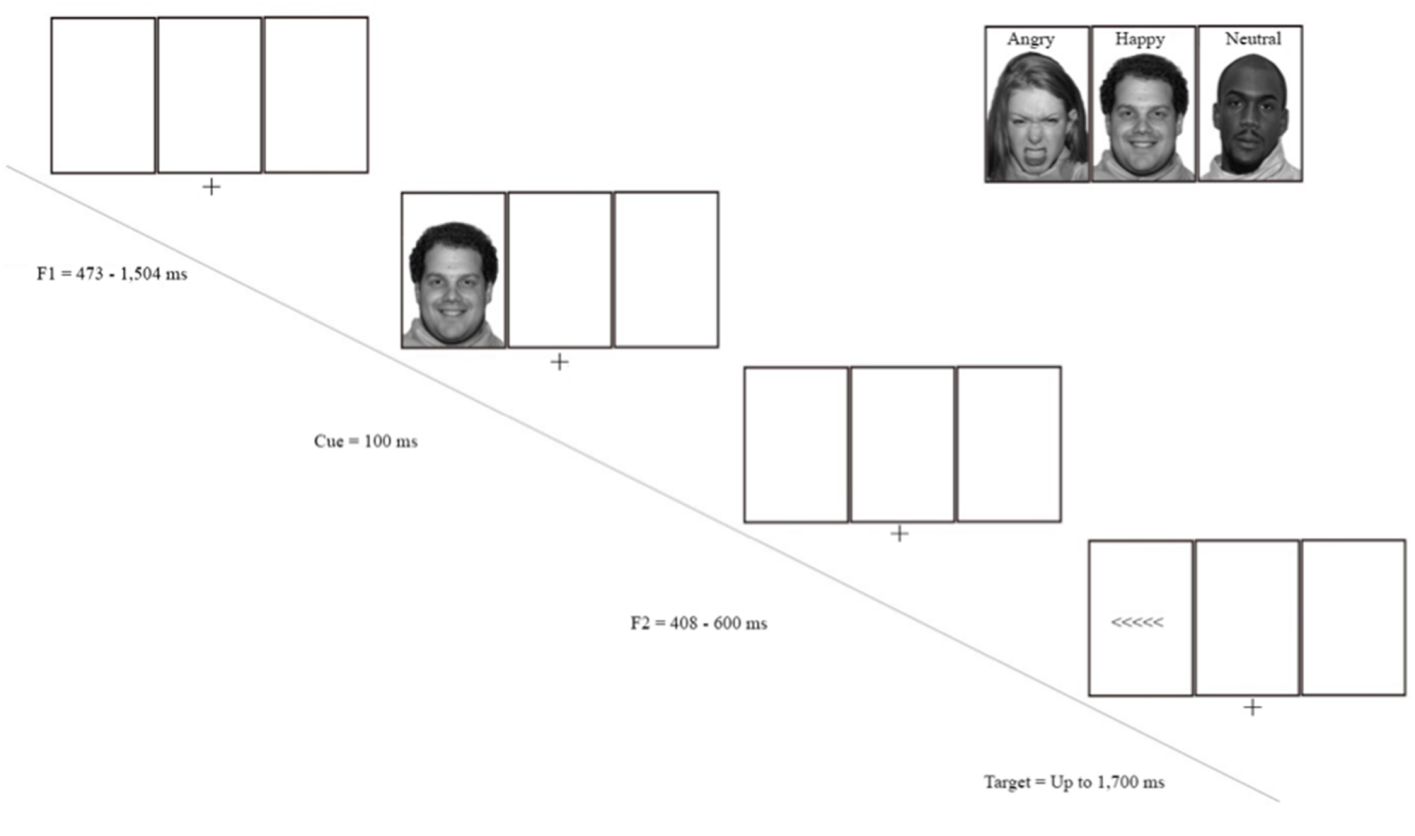
Experimental procedure of a valid trial and face stimuli.

The face-cued flanker task was presented in nine blocks (three blocks for each emotional face type), with 96 trials per block for a total of 864 trials. Participants were given a 16-trial practice block before starting the nine experimental blocks and were given feedback on their performance. Two versions (counterbalanced) were administered in which either the angry face block or happy face block appeared first. Within a block, trials were randomly presented and consisted of the following: 16 no cue trials, 16 center cue trials, 48 valid spatial cue trials, and 16 invalid spatial cue trials. Valid trials were disproportionately more frequent compared to invalid trials in order to limit the anticipation of an invalid cue. Fig 1 depicts the sequence of events in each trial prior to the flanker task. Responses were made to the flanker task by pressing the left or right mouse button, depending on the direction in which the central arrow was pointing. No feedback on performance was given during experimental blocks. Participants were given short breaks in-between each of the nine blocks and the entire task took approximately seventy minutes to complete. Only correct trials were included in analyses.

### EEG recording and data reduction

EEG activity was recorded continuously via 64 Ag/AgCl scalp electrodes embedded in an elasticized nylon cap (BioSemi; Amsterdam, NL). Electrodes in this system are arranged according to the international 10/20 system. Eye movements were monitored by electro-oculogram (EOG) using four flat-type facial electrodes placed one cm above and below the left eye (vertical eye movements) and one cm to the outer corner of each eye (horizontal eye movements). Electrodes used within this study preamplify the EEG signal in order to improve the signal-to-noise-ratio. EEG was recorded at a sampling rate of 512 Hz. During EEG acquisition, the voltage from each electrode was referenced online with respect to the common mode sense active electrode and the driven right leg electrode which produces a monopolar (nondifferential) channel.

Offline data processing was conducted using Brain Vision Analyzer (Version 2.2, GmbH; Munich, DE). The continuous EEG data was filtered with a high pass frequency of .1 Hz and a low pass frequency of 30 Hz and re-referenced offline to an average reference. To examine face cue processing, face cue- locked data were baseline corrected using 200 ms prior to stimulus presentation and segmented between -200ms and 600ms for each trial.

Data were corrected for blinks using independent components analysis (ICA). Artifacts were identified using the following criteria: voltage steps that were greater than 75μV, amplitude differences greater than ±105 μV within a segment, and activity lower than .2μV within a 400 ms interval and maximum amplitude differences greater than 100μV within an entire segment were considered artifacts and excluded from analyses. After ICA, data were visually inspected to confirm successful eye blink removal and to detect any remaining artifacts. Data from individual channels containing artifacts were rejected on a trial-by-trial basis. ERPs were generated by identifying the mean amplitude between 90 ms and 150 ms for the P1 and the mean amplitude between 140 ms and 200 ms for the N170 post face cue presentation. The time windows and electrodes chosen for both the P1 and N170 were based on visual inspection of the topographical distribution of the grand averaged data. ERPs used in analyses were computed by averaging across the following electrodes: **P1** (P5, P6, P7, P8, PO7, PO8) and **N170** (P5, P7, P6, P8, CP5, CP6). Of the possible 48 center cue trials, 144 valid cue trials, and 48 invalid cue trials per face type, the average number of artifact-free EEG trials were: P1 [angry center: (*M* = 46.36, *SD* = 3.57) valid: (*M* = 139.51, *SD* = 8.00) invalid: (*M* = 46.70, *SD* = 3.04); happy center: (*M* = 46.57, *SD* = 2.22) valid: (*M* = 139.82, *SD* = 7.39) invalid: (*M* = 46.27, *SD* = 2.56); neutral center: (*M* = 45.65, *SD* = 3.91) valid: (*M* = 137.39, *SD* = 11.64) invalid: (*M* = 45.65, *SD* = 3.81)] and N170 [angry center: (*M* = 46.03, SD = 3.54) valid: (*M* = 138.82, *SD* = 8.26) invalid: (*M* = 46.41, *SD* = 3.15); happy center: (*M* = 46.93, SD = 1.79) valid: (*M* = 140.62, *SD* = 5.97) invalid: (*M* = 46.50, *SD* = 2.02); neutral center: (*M* = 46.03, *SD* = 3.85) valid: (*M* = 138.64, *SD* = 11.16) invalid: (*M* = 45.99, *SD* = 3.76)]. Mean amplitudes were calculated separately for each face condition (angry, happy, neutral) using all artifact-free trials.

## Results

### Descriptive statistics

Table 1 presents mean response times and standard deviations for congruent and incongruent flanker trials, by cue type and emotion type. Table 2 presents executive attention scores for all cue and emotion types. Table 3 presents the mean P1 and N170 amplitudes and standard deviations for each face and trial type respectively.

**Table 1:** Means and standard deviations for response times to congruent and incongruent trials by cue type and emotion type (ms).

	Valid	Invalid	Center	No Cue	Angry	Happy	Neutral
Congruent	526.85 (69.34)	614.80 (72.10)	585.49 (74.78)	641.41 (79.43)	601.18 (108.04)	595.45 (78.44)	597.86 (86.84)
Incongruent	675.49 (82.27)	801.90 (97.81)	766.94 (97.83)	812.13 (91.59)	767.93 (114.33)	769.71 (99.26)	763.60 (91.65)

*Note*: Standard deviations are presented in parentheses.

**Table 2:** Executive attention scores for each cue and face trial type (ms).

	Valid	Invalid	Center	No Cue
Happy	145.80 (75.09)	182.08 (81.71)	169.07 (78.03)	180.23 (94.20)
Angry	142.24 (78.08)	182.37 (73.80)	184.78 (74.04)	153.15 (109.63)
Neutral	146.97 (54.95)	186.38 (66.11)	180.82 (61.57)	162.68 (85.41)

*Note*: Executive attention score are calculated by subtracting the mean response time of the congruent flanker trials from the mean response time of the incongruent flanker trials. Standard deviations are presented in parentheses.

**Table 3:** Means and standard deviations for P1 and N170 amplitudes by cue type and emotion type (μV).

P1		Valid	Invalid	Center
	Angry	1.52 (1.09)	1.79 (1.41)	2.21 (1.66)
	Happy	1.63 (1.43)	1.75 (1.59)	1.76 (1.73)
	Neutral	1.55 (1.25)	1.35 (1.60)	1.46 (1.69)
N170		Valid	Invalid	Center
	Angry	-0.13 (1.39)	-0.12 (1.54)	-0.85 (1.54)
	Happy	0.03 (1.50)	0.17 (1.27)	-0.71 (1.67)
	Neutral	0.21 (1.45)	0.25 (1.61)	-0.32 (1.47)

*Note*: Standard deviations are presented in parentheses.

### Effects of emotion and cue validity on conflict interference

First, to test the hypothesis that emotional context would influence the effect of cue validity on conflict interference, we conducted a 4 (Cue Type: valid, invalid, center, no cue) X 3 (Emotional Face Type: angry, happy, neutral) repeated measures ANOVA. Specifically, we predicted that valid versus invalid cues would lead to reduced conflict interference (superior executive attention efficiency), but only for happy and neutral trials; the advantage conferred by valid cues would be reduced when cues were angry faces.

This analysis yielded a main effect of Cue Type, *F*(3,69) = 8.92, *p* < .001, partial *η*^*2*^ = .28. As predicted, conflict interference was reduced followed valid (*M* = 145.00, *SD* = 55.09) compared to invalid cues (*M* = 183.61, *SD* = 64.78), *t*(23) = -5.35, *p* < .001, Cohen’s *d* = .64 and compared to the comparison cues, center (*M* = 178.22, *SD* = 57.91), *t*(23) = -4.54, *p* < .001, Cohen’s *d* = .09 and no cues (*M* = 165.35, *SD* = 78.52), *t*(23) = -2.63, *p* < .05, Cohen’s *d* = .25. No other significant effects emerged, and contrary to predictions, emotion did not influence cue validity effects.

### Effects of emotion cue type on ERPs

To test the prediction that P1 and N170 amplitudes would be larger to emotional versus neutral stimuli, a 3 (Cue Type: valid, invalid, center) X 3 (Emotional Face Type: angry, happy, neutral) X 2 (Hemisphere: left, right) repeated measures ANOVA was conducted for each ERP component. Greenhouse-Geisser was used to correct for sphericity assumption violations. Although we had no specific hypotheses regarding differences between valid, invalid, and center cues, we explored possible interactions with Emotion Face Type by retaining Cue Type as a within-subject variable.

#### P1

**Fig 2:**
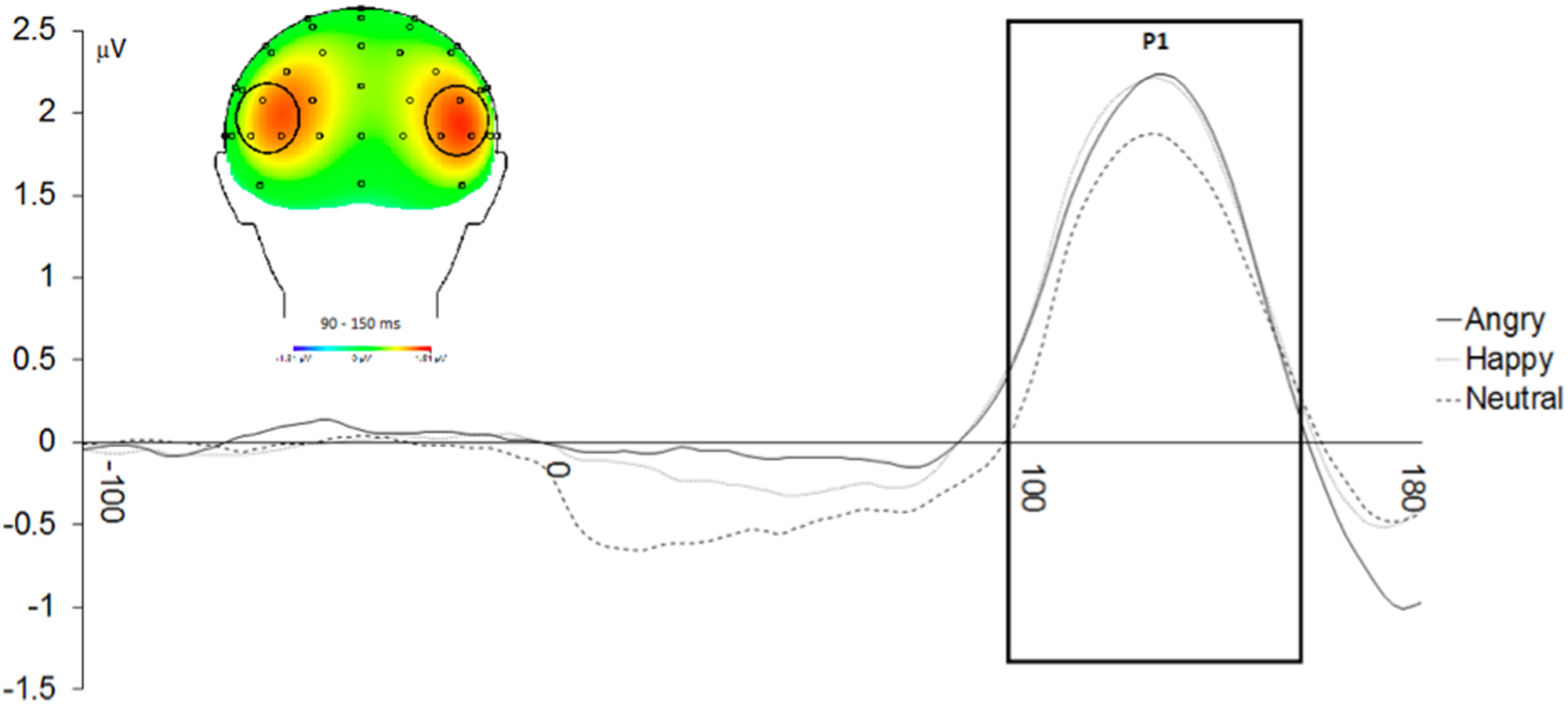
Enhanced P1 amplitudes to angry and happy versus neutral face cues. The P1 was quantified as the mean amplitude from 90 ms to 150 ms at P5, P6, P7, P8, PO7, and PO8.

As predicted, there was a main effect of Emotional Face Type, *F*(2,48) = 5.39, *p* < .01, partial *η*^*2*^ = .18, in which P1 amplitudes to angry faces (*M* = 1.84, *SD* = 1.29) were greater than to neutral faces (*M* = 1.46, *SD* = 1.35), *t*(24) = 3.015, *p* < .01, Cohen’s *d* = .29. In addition, P1 amplitudes to happy faces (*M* = 1.71, *SD* = 1.48) were greater than to neutral faces (*M* = 1.46, *SD* = 1.35), *t*(24) = 2.031, *p* = .054, Cohen’s *d* = .18 (Fig 2). No other significant effects emerged.

#### N170

**Fig 3:**
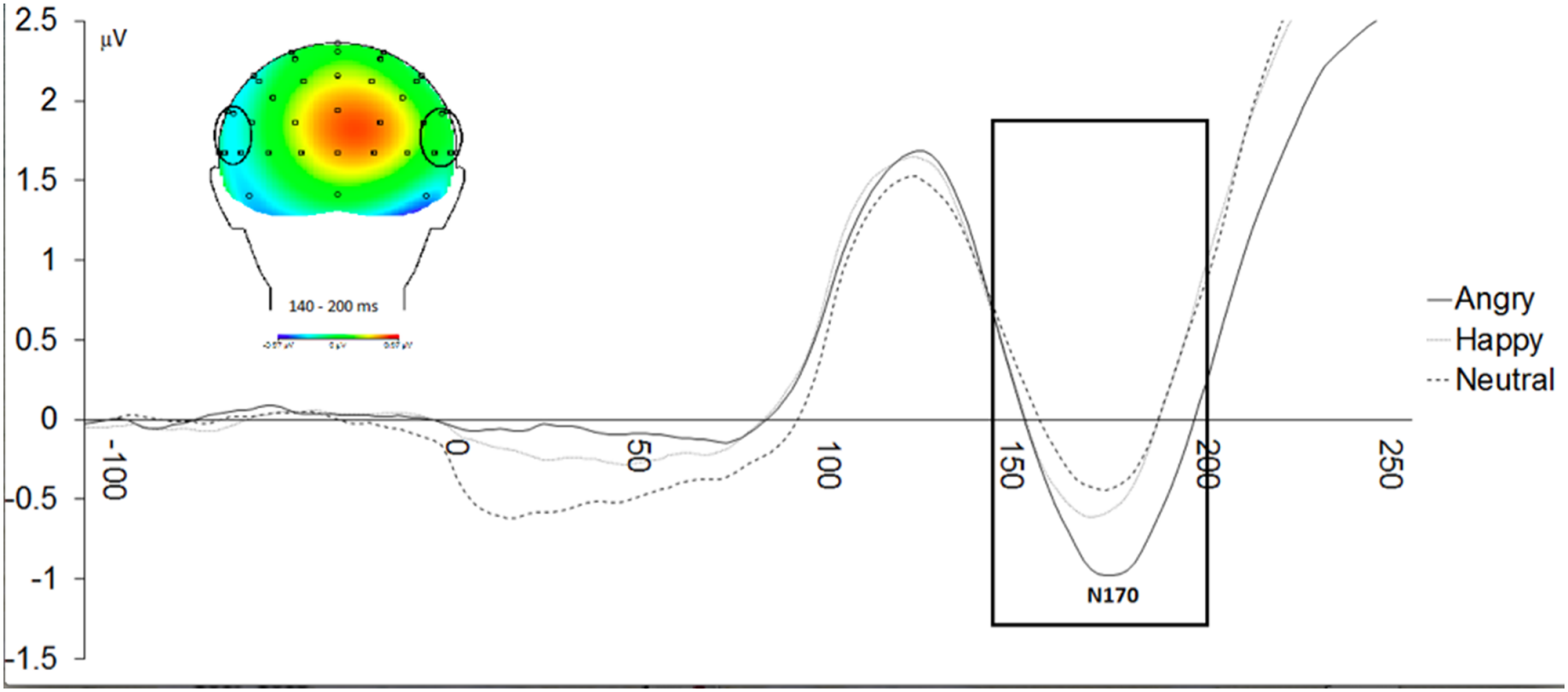
N170 amplitudes were larger to emotional (angry and happy) versus neutral faces; however amplitudes were also greater for angry versus happy faces. The N170 was quantified as the mean amplitude from 140 ms to 200 ms at P5, P7, P6, P8, CP5, and CP6.

Similar to the P1, there was a main effect of Emotional Face Type, *F*(1.51,36.36) = 7.07, *p* < .01, partial *η*^*2*^ = .23. As predicted, N170 amplitudes to angry faces (*M* = -.37, *SD* = 1.40) were greater than to happy faces (*M* = -.17, *SD* = 1.37), *t*(24) = -2.24, *p* < .05, Cohen’s *d* = .14 and to neutral faces (*M* = .05, *SD* = 1.31), *t*(24) = -3.01, *p* < .01, Cohen’s *d* = .31. In addition, N170 amplitudes to happy faces (*M* = -.17, *SD* = 1.37) were greater than to neutral faces (*M* = .05, *SD* = 1.31), *t*(24) = -2.20, *p* < .05, Cohen’s *d* = .16 (Fig 3). No other significant effects emerged.

### Individual differences in sensitivity to emotional face types

Given the sensitivity of the P1 and N170 to emotion, we next examined the impact of individual differences in emotional processing to predict how emotion effects cue validity. In other words, individuals showing enhanced ERP responses to happy faces should be particularly sensitive to positive emotional cues and show significantly facilitated performance. Similarly, individuals showing enhanced processing of angry faces should evidence a disruption in performance following angry cues, and show dampened validity effects on performance. Difference scores were computed separately for the P1 and N170 to assess the degree to which individuals showed greater attentional allocation and dicrimination of emotional versus the control condition of neutral faces (e.g. happy—neutral and angry—neutral). Then, participants were categorized into high amplitude and low amplitude groups for each difference score using a median split (see Table 4). Scores falling below the median (more negative for N170) were placed in the high amplitude group while scores falling above (less negative for N170) made up the low amplitude group. For the P1, the median value for the angry—neutral difference score was .327 and for the happy—neutral difference score was .114. Sixteen individuals were consistently either high or low across both emotions, whereas 9 individuals fell into different categories for each emotion. For the N170, the median value for the angry—neutral difference score was -.433 and for the happy—neutral difference score was -.358. Thirteen individuals were consistently either high or low across both emotions, whereas 12 individuals fell into different categories for each emotion. The cue comparison conditions were left out of these analyses in order to focus on effects of validity and because ERPS were not generated during no cue trials.

**Table 4:** Means and standard deviations for N170 amplitudes by high and low groups (μV).

	High	N	Low	N
Angry	-.852 (.538)	13	.054 (.519)	12
Happy	-.545 (.197)	13	.131 (.498)	12

Note: Standard deviations are presented in parentheses.

A series of 2 (Cue Type: valid, invalid) X 3 (Emotional Face Type: angry, happy, neutral) mixed ANOVAs were conducted with either high or low P1 or high and low N170 groups entered as the between-subjects variable. Conflict interference was the dependent variable. For the high versus low groups, we predicted that those showing greater P1 and N170 to *angry faces* (the high group) would show reduced cue validity effects, measured as greater conflict interference (reduced executive attention performance) following valid versus invalid cues. Conversely, for the high versus low groups, we predicted that those showing greater P1 and N170 to *happy faces* would show stronger cue validity effects, measured as reduced conflict interference (enhanced executive attention performance) following valid versus invalid cues.

#### P1

No significant effects emerged using P1 low and high amplitude groups.

#### N170 to angry faces

**Fig 4:**
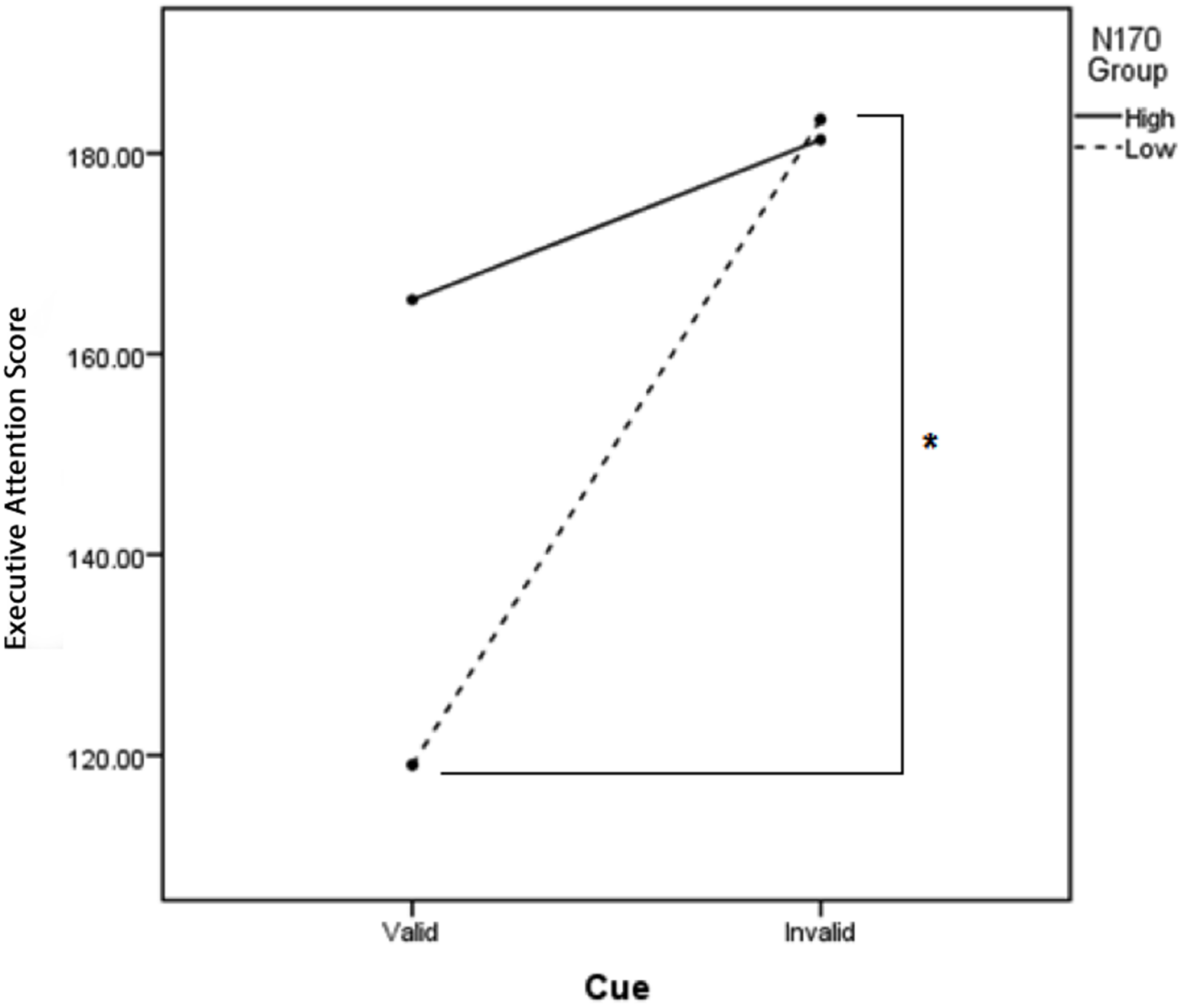
Participants in the low-N170 angry group performed significantly better on valid angry-cued trials compared to invalid trials. In contrast, participants in the high-N170 angry group executive attention performance was not enhanced on valid compared to invalid angry-cued trials.

There was a significant three-way interaction of Emotional Face Type X Cue Type x N170-Angry Group, *F*(2, 44) = 4.614, *p* = .015 partial *η*^*2*^ = .17. No between-group differences reached significance. However, examination of within group differences revealed that the low N170-angry group showed a significant validity effect—that is, they evidenced significantly lower conflict interferences scores (better executive attention) for valid (*M* = 119.06, *SD* = 91.24) compared to invalid angry face cues (*M* = 183.37, *SD* = 72.76), *t*(10) = -2.97, *p* = .013, Cohen’s *d* = .78. Benjamini-Hochberg adjusted p value at a FDR of 10% is .013(6/1) or .078. If the adjusted p value is smaller than the false discovery rate, the test is significant. Conversely, as predicted, in the high N170-angry group, the validity effect was no longer significant: that is, there was not a significant conflict interference difference between valid (*M* = 165.41, *SD* = 57.01) compared to invalid angry face cues (*M* = 181.37, *SD* = 78.05), *t*(11) = -1.29, *p* = .23, Cohen’s *d* = .23; see Fig 4. Taken together, these results illustrate that the beneficial effect of cue validity on executive attention performance was specifically disrupted following angry faces in the high N170-angry group.

#### N170 to happy faces

**Fig 5:**
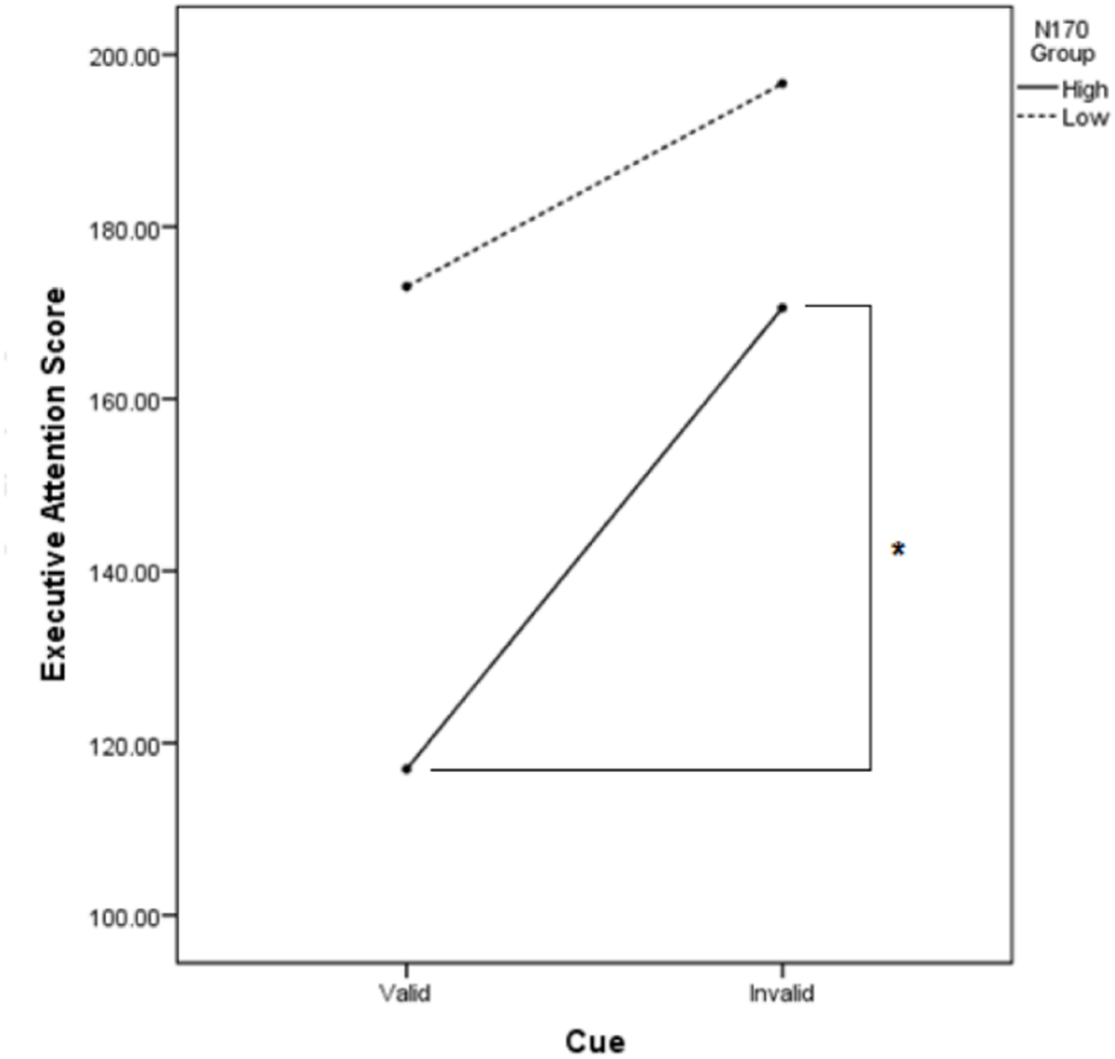
The high-N170 happy group showed significantly better executive attention performance (less conflict interference) on all valid compared to invalid cue trials.

There was a significant interaction between Cue Type and N170-Happy Group, *F*(1, 22) = 5.10, *p* = .03 partial *η*^*2*^ = .19. Follow-up independent-samplest-tests indicated that the high versus low N170-happy group showed reduced conflict interference on valid cue trials across all Emotional Face Type conditions (*M* = 116.97, *SD* = 54.04 vs. *M* = 173.04, *SD* = 41.35, respectively), *t*(22) = -2.86, *p* = .01, Cohen’s *d* = 1.17; see Fig 5. Benjamini-Hochberg adjusted p value at a FDR of 10% is .01(3/1) or .03. If the adjusted p value is smaller than the false discovery rate, the test is significant. The two groups did not significantly differ on invalid cue trials. No other comparisons reached significance.

## Discussion

The present study addressed a significant gap in our understanding of how emotion and cue validity interact and affect attention performance. Conflict interference was significantly reduced on valid cue trials as compared to invalid trials. Therefore, as expected, performance was enhanced when face cues were presented in the same location as the target stimulus for our sample overall. This is consistent with the plethora of previous research documenting the effects of cue validity on attention [3, 2, 5, 4]. Contrary to predictions, conflict interference was not significantly different following positive and negative emotional face cues. However, effects of emotion did emerge when individual differences in neurocognitive responses to emotional faces were examined, suggesting a role for such individual differences in examining the impact of emotion on cue validity.

Although previous findings have been mixed in terms of the sensitivity of the P1 and N170 to emotion, type [67, 63], we found evidence to support the differential sensitivity of these components to positive, negative, and neutral faces. Specifically, P1 and N170 amplitudes were larger to angry and happy faces versus neutral faces. In addition, the N170 was sensitive to valence, with the magnitude of the N170 being larger to angry versus happy faces. This emotional sensitivity of target ERPs lends credence to our approach of using them as a measure of cognitive-emotional individual differences.

We specifically targeted early-emerging ERPs reflecting two discrete stages of attention and visual processing: attention allocation (P1; [47]), and face-sensitive visual discrimination (N170; [60, 54]). Thus, it is not surprising that individual differences were seen at the point of stimulus-sensitive visual discrimination (N170). Although the mere presence of emotional cues did not influence cue validity, individual neurocognitive responses did. Individuals in the highN170 angry group showed specific disruptions in executive attention performance when cues were valid angry faces, compared to individuals in the low-N170 angry group and the total sample. Conversely, participants in the high-N170 happy group showed facilitated attention on valid cue trials across all face types, suggesting a more generalized performance advantage. Such findings are consistent with previous studies documenting effects of physiological effects of face processing in the absence of differences in behavioral performance [72, 73, 74]. Results also add to the body of research documenting the link between emotional face cue processing as measured by ERPs and attention performance via reaction times [6, 70]. Findings are also consistent with previous models, such as that of Huntsinger [32], positing that positive and negative emotions can either minimize or broaden attentional focus depending on context and task demands. Taken together, previous notions of a fixed relationship between emotion and attention may underestimate the role of individual differences, particularly those reflecting implicit neurocognitive responses to emotional stimuli and context.

To manipulate cue validity behaviorally, the current study alternated valid and invalid cues in order to draw attention towards or away from the target stimulus. This is similar to global/local attentional priming paradigms, such as that used by Huntsinger [34], which showed that flanker performance varied by emotional context (mood induction) but only after attentional focus was primed as well. Interestingly, we also did not find performance effects until individual differences in neurocognitive responses to emotion were taken into account. In that sense, ERPs may provide sensitivity of measurement and predictive power in the absence of priming.

The present findings further suggest that the effects of emotion on attention are likely not uniform across all groups of people. For example, Amir et al. [75] found that in a clinical sample of socially-anxious individuals, reaction times detecting a target probe following invalid cues were significantly slower, but only when cues were social threat words. Response times following neutral and positive word cues, however, were not slower. Subsequent studies should evaluate performance differences in clinically-anxious samples to explore the potential use of biomarkers like the N170 to identify dysfunctional patterns and emotional sensitivities associated with anxiety and depression. Moreover, an additional important future research direction is to systematically vary the degree to which emotional faces are relevant within an attention task (e.g., primes, cues, or distracters) in order to directly examine the role of task-relevance on emotion-attention interactions.

One limitation of the design is that valid and invalid cues were not presented at an equal frequency. However, this is a necessary design feature in order to manipulate validity (at least 75% vs. 25% for valid and invalid cues). As a result, the reported findings should be considered in terms of both differences in validity and in the disproportion of trial types. An additional limitation in interpreting the present findings is that results are based on small groups. Although a substantial number of previous ERP studies have similar sample sizes (see [66, 45]) and have divided their original sample into smaller groups based on performance in order to evaluate individual differences in ERPs (see [76, 77]), ideally a larger sample would provide more confidence in significant group differences. Lastly, future studies should explore later stages of emotional processing and attention using later-emerging ERP components such as the N2 and P3 [44, 45]. Another interesting future direction will be to evaluate error rates and concurrent response-locked brain activity via the error-related negativity (ERN) and error positivity (Pe), to examine another dimension of emotional cue validity effects [43].

## Conclusions

Collectively, findings suggest that individual differences in neurocognitive responses to emotional cues influence basic cue validity effects on attention performance. Moreover, findings are consistent with models that highlight a flexible, context-sensitive relationship between emotion and attention [28, 32, 70] and suggest that emotions may not directly tune the scope of attention in the same way for each person. This perspective builds on previous theories highlighting specific cognitive processes associated with negative [78, 79] and positive [16] affect, such as the broadening and narrowing of attention, but further highlight the role of neurocognitive individual differences in understanding the complex interplay between emotion and attention.
