# Correction: Emotional cue validity effects: The role of neurocognitive responses to emotion

**DOI:** 10.1371/journal.pone.0189560

**Published:** 2017-12-07

**Authors:** The PLOS ONE Staff

There is an error in affiliation 3 for author Amishi Jha. Affiliation 3 should be: University of Miami, Coral Gables, Florida, United States of America. The publisher apologizes for the error.
